# Cytokines can counteract the inhibitory effect of MEK-i on NK-cell function

**DOI:** 10.18632/oncotarget.11504

**Published:** 2016-08-22

**Authors:** Claudia Manzini, Roberta Venè, Irene Cossu, Marina Gualco, Simonetta Zupo, Mariella Dono, Francesco Spagnolo, Paola Queirolo, Lorenzo Moretta, Maria Cristina Mingari, Gabriella Pietra

**Affiliations:** ^1^ IRCCS Istituto Giannina Gaslini, Genoa, Italy; ^2^ Oncologia Molecolare e Angiogenesi, IRCCS AOU San Martino-IST, Genoa, Italy; ^3^ Anatomia Patologica, IRCCS AOU San Martino-IST, Genoa, Italy; ^4^ Diagnostica Molecolare, IRCCS AOU San Martino-IST, Genoa, Italy; ^5^ Oncologia Medica 2, IRCCS AOU San Martino-IST, Genoa, Italy; ^7^ IRCCS Ospedale Pediatrico Bambino Gesù, Roma, Italy; ^8^ Department of Experimental Medicine, University of Genoa, Genoa, Italy; ^9^ Immunologia, IRCCS AOU San Martino-IST, Genoa, Italy; ^10^ Center of Excellence for Biomedical Research, University of Genoa, Genoa, Italy

**Keywords:** NK cells, melanoma, BRAF/MEK inhibitors, immunotherapy, cytokines, Immunology and Microbiology Section, Immune response, Immunity

## Abstract

Oncogene-targeted therapies based on mutated BRAF- and/or MEK-specific inhibitors have been developed for melanoma treatment. Although these drugs induce tumor regression in a high percentage of patients, clinical responses are frequently limited in time and tumors often recur. Recent studies suggested that the combination of BRAF/MEK inhibition with immunotherapy could represent a promising strategy for the cure of melanoma. NK cells are suitable effectors for tumor immunotherapy. Here we show that PLX4032 (a mutant BRAF^V600^ inhibitor) had no effect on the functional properties of NK cells cultured in the presence of IL-2 or IL-15. In contrast, PD0325901 (a MEK inhibitor) induced the down-regulation of the main activating NK receptors and inhibited NK cell function. Importantly, PD0325901 did not affect the anti-tumor activity of NK cells that had been exposed to a combination of IL-15 and IL-18. In addition, both PLX4032 and PD0325901 did not exert any inhibitory effect on *in vitro* IL-2 or IL-15 pre-activated NK cells.

Our data may provide a rationale for future clinical protocols that combine IL-15/IL-18 cytokine administration with MEK inhibitors. In addition, they suggest that oncogene-targeting drugs are compatible with NK-based adoptive therapy.

## INTRODUCTION

Melanoma is a major world health problem with an incidence that is increasing at a rate faster than any other solid malignancy. The overall survival rate for patients with metastatic melanoma is poor and new forms of treatment are clearly needed. Approximately 50% of cutaneous melanomas harbor a somatic mutation in the gene encoding BRAF, a serine/threonine protein kinase that activates the mitogen-activated protein kinase (MAPK)/ERK-signaling pathway [1]. Among the BRAF mutations detected in melanoma, over 80% are represented by a single nucleotide mutation characterized by a glutamic acid for valine at codon 600 (BRAF^V600E^). BRAF mutations lead to constitutive activation of the MAPK signaling pathway involved in different mechanisms of tumor progression including evasion of apoptosis, unchecked cell replication, neoangiogenesis, tissue invasion, metastasis as well as escape from immune response [2].

The identification of BRAF mutations offered an opportunity to test novel oncogene-targeted therapies for melanoma. In particular, PLX4032 (a highly active agent for the treatment of BRAF^V600^ mutant melanoma) has been found to induce tumor regression in high percentages of patients with BRAF^V600E^-positive metastatic melanoma. However, clinical responses were limited in time (less than 12 months) in most patients [3]. The emergence of resistance may reflect reactivation of MAPK pathway due to new mutations [4–6] or activation of alternative pathways involving MAPK and PI3K/Akt [7, 8]. In particular, analysis of clinical specimens and cell lines resistant to the selective BRAF inhibitor (BRAF-i) allowed to identify a variety of molecular mechanisms that reactivate signaling *via* MEK and ERK. In view of these limitations, new protocols have been designed in which BRAF-targeted therapies have been associated with MEK inhibitors (MEK-i), such as Trametinib [9] or Cobimetinib [10]. Since also immunotherapy may induce long lasting responses [11], an area of ongoing investigation involves the combination of BRAF-i/MEK-i with immune-based therapies. However, the efficacy of cell-based immunotherapy, due to the potent anti-tumor activity of both cytolytic T lymphocytes (CTL) and natural killer (NK) cells, may be compromised by the simultaneous use of oncogene-targeted therapies. In this context, in order to efficiently combine kinase inhibitors with immunotherapy, it is critical to assess whether these drugs may influence the effector cell responses. It has been shown that inhibition of the MAPK pathway using PLX4720 (a selective inhibitor of BRAF^V600E^) did not affect the viability and function of T cells. In addition, it induced an increased expression of melanocyte differentiation antigens (MDAs), thus conferring a more potent antigen-specific cytotoxicity to CTL [12]. Other studies showed that BRAF inhibition resulted in an improved infiltration of adoptively-transferred T cells *in vivo*, thus enhancing the anti-tumor activity of adoptive cell transfer (ACT) therapy [13]. In contrast, MEK inhibition had a similar effect on MDAs [12], but affected multiple functions of *in vitro* isolated T-lymphocytes [12, 14]. However, in contrast with *in vitro* data, *in vivo* studies suggest that MEK-i do not interfere with the anti-tumor activity of T-cell-based therapy [15] or of specific immunomodulatory antibodies targeting PD-1, PD-L1 and CTLA-4 [16]. Recently, it has been demonstrated that *in vivo* MEKi, when used in combination with PD-L1 checkpoint blockade, potentiate T-cell-mediated anti-tumor immunity by increasing the frequency of intratumoral antigen-specific effector CD8^+^ T cells [17].

Besides specific T lymphocytes, it is now well established that also NK cells play a role in cancer immune-surveillance. Indeed, individuals with high NK cell activity have been shown to display a reduced risk of developing cancer [18]. In addition, in different human and murine tumors, a high level of NK cell infiltration correlates with a better prognosis [19–21].

The process of NK cell activation is the result of a fine balance between signals mediated by an array of triggering and inhibitory surface receptors [22–24]. The NK cell receptors involved in tumor cell killing include the HLA class I-specific inhibitory receptors (i.e. KIRs and CD94/NKG2A) and major activating NK receptors (including NKp30, NKp46, NKp44, NKG2D and DNAM-1). In the absence of inhibitory signals the interaction between activating receptors and their specific ligands on tumor cells results in NK cell triggering and target cell lysis. The main ligands of activating NK receptors include MICA/B, ULBPs (recognized by NKG2D) [25, 26] Nectin-2 and PVR (recognized by DNAM-1) [27], B7H6 (recognized by NKp30) [28, 29] and a novel isoform of the mixed-lineage leukemia-5 protein (MLL5) (recognized by NKp44) [30]. In most instances, these ligands are not (or only marginally) expressed by normal resting cells while they become highly expressed on tumor cells. It has been shown that melanoma cells are susceptible to lysis by IL-2-activated NK cells. This effect is consequent both to down-regulation of MHC class I antigens and to the expression of ligands of activating NK receptors on tumor cells.

The actual efficacy of combination treatments involving MAPK inhibitors and NK cell-based immunotherapy, as well as the occurrence of possible interference with NK cell function, remains to be fully clarified. A study in mice showed a significant enrichment in intratumoral NK1.1^+^ NK cells after treatment with the BRAF-i PLX4720 [31]. Along this line, murine NK cells have been shown to play a critical role in favoring the anti-metastatic effect of BRAF inhibitors [32]. However, limited information is available on whether BRAF-i and MEK-i may directly affect human NK cells [32, 33].

In this study, we show that PLX4032, a selective BRAF-i, has no inhibitory effect either on NK cell proliferation in response to cytokines (including IL-2, IL-15, and IL15 plus IL-18) or on NK cell function (cytotoxicity and cytokine production). PD0325901 has a negative effect on NK cells exposed to IL-2 and IL-15, but not on NK cells treated with IL-15/IL-18. In view of the possibility to combine adoptive immunotherapy with *in vitro* expanded NK cells and BRAF-i and/or MEK-i, we further evaluated their possible interference with cytokine-pre-activated NK cells. Importantly, our data indicate that both BRAF-i and MEK-i do not exert any inhibitory effect on both IL-2- and IL-15- pre-activated NK-cells.

Thus, our study provides a clue for designing future therapies that combine IL-15/IL-18 cytokine administration and/or NK cell-based immunotherapy with kinase-targeted agents.

## RESULTS

### Effect of BRAF-i and MEK-i on NK cell survival

**Figure 1:**
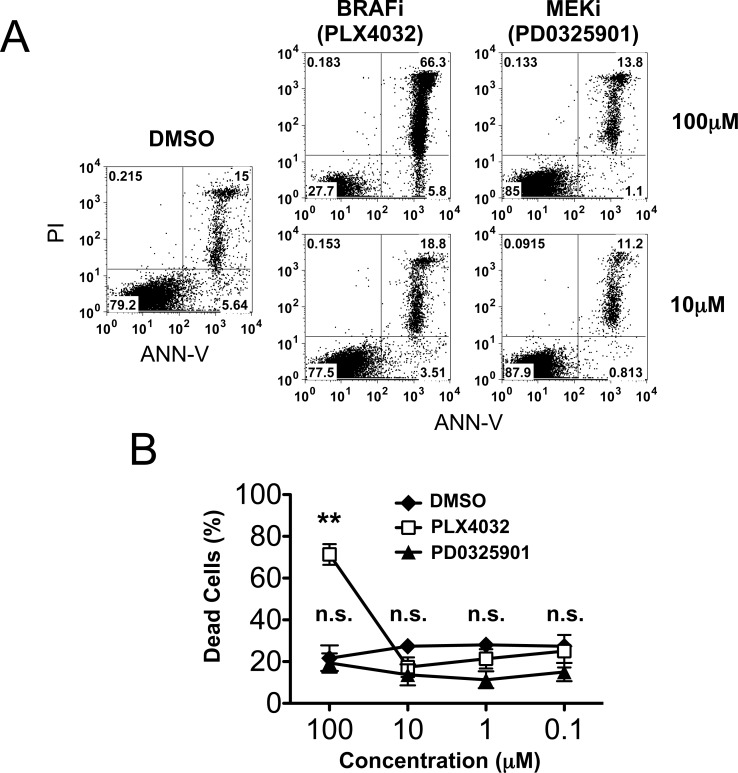
Survival of NK cells exposed to increasing concentrations of either BRAF-i or MEK-i Percentage of apoptotic/necrotic cells upon treatment with PLX4032 or PD0325901. NK cells obtained from 3 healthy donors were cultured for 5 days with IL-2 in the presence of different concentrations (100μM, 10μM, 1μM and 0.1μM) of the drugs. NK cells cultured with IL-2 in the presence of DMSO represent the negative controls. **A.** Representative flow cytometric analysis based on ANN-V and PI staining of NK cells either untreated (DMSO) or treated with PLX4032 or PD0325901 (100μM and 10μM). Numbers indicate the percentage of cells in each quadrant. **B.** Percentage of apoptotic/necrotic (ANN-V^+^ PI^−^, ANN-V^+^PI^+^, and ANN-V^−^PI^+^) NK cells either untreated (DMSO) or exposed to the indicated drug concentrations. Results are obtained from 3 independent experiments. Each point represent mean ± SD. **, *p* < 0.01; n.s., not significant by two tailed paired Student's *t* test.

We first analyzed the effect of selective inhibitors of BRAF (PLX4032) or MEK (PD0325901) on NK cell viability. To this end, NK cells, freshly isolated from peripheral blood (PB) of healthy donors, were cultured in IL-2 in the presence of PLX4032 and PD0325901 at 4 different drug concentrations (100μM, 10μM, 1μM, and 0.1μM). After 5 days, the percentages of early apoptotic (Annexin V^+^/PI^−^), late apoptotic (Annexin V^+^/PI^+^), and necrotic cells (Annexin V^−^/PI^+^) were evaluated in both treated and untreated cultured NK cells. Annexin V/PI staining revealed a markedly different pattern of susceptibility of NK cells to BRAF-i and MEK-i. As shown in Figure 1A, NK cells cultured in the presence of high concentrations (100μM) of PLX4032 showed a poor viability, whereas those treated with 100μM PD0325901 were not significantly affected. On the other hand, 10μM or lower concentrations (1μM-0.1μM) of either BRAF-i or MEK-i did not affect NK cell viability (Figure 1A, and data not shown). Figure 1B shows the statistical analysis of the experiments performed using NK cells isolated from 3 different donors and treated with graded concentrations (100μM, 10μM, 1μM, and 0.1μM) of PLX4032 or PD0325901. These results show that NK cells survive up to 10μM concentrations of BRAF-i and MEK-i.

### Effect of BRAF-i and MEK-i on the expression of activating NK receptors

**Figure 2:**
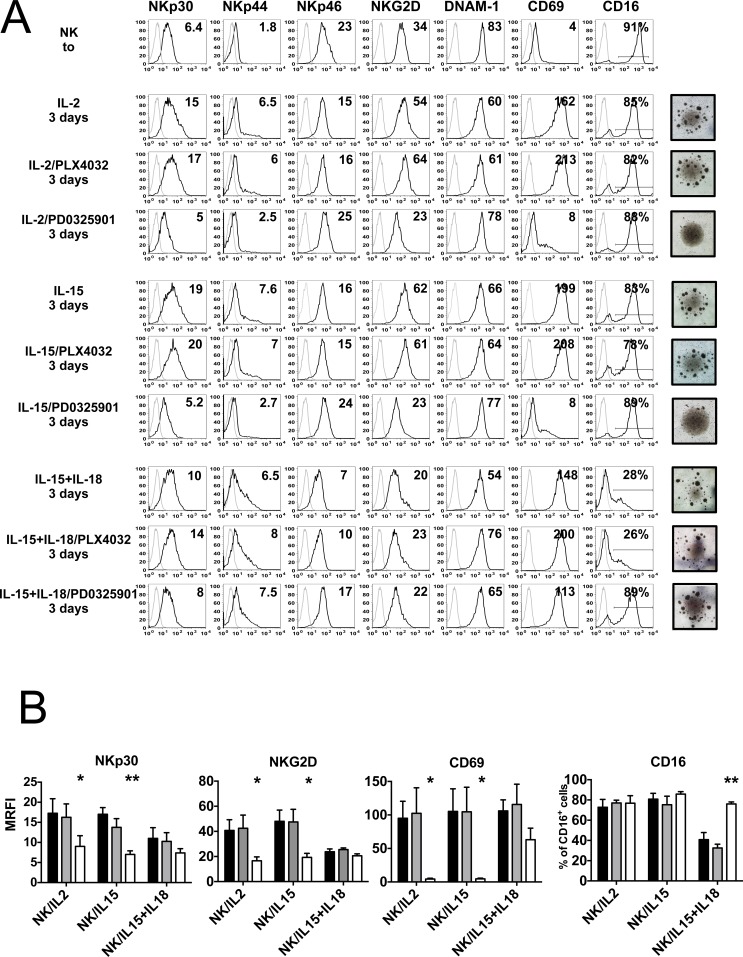
Effect of BRAF-i and MEK-i on NK cell receptor expression Surface phenotype of NK cells isolated from 4 healthy donors cultured for 3 days with IL-2, IL-15 or IL15/IL-18 either in the absence (DMSO) or in the presence of PLX4032 and PD0325901 (10 μM). **A.** Expression of the main activating receptors (black histograms) was analyzed by fluorescence-activated cell sorting on NK cells freshly isolated (t0) or cultured (day 3) with the indicated cytokines either in the absence or in the presence of the drugs. Gray profiles represent negative controls. Numbers indicate MRFI (or the % of CD16^+^ NK cells). A representative experiment out of 4 performed is shown. The morphological aspect of NK cells upon treatment with the indicated drugs is shown by light microscopy photos on the right (Microscope Leica DM LB2) **B.** Statistical analysis of the expression of NKp30, NKG2D, CD69 and CD16 on NK cells cultured either alone (black bars) and in the presence of PLX4032 (gray bars) or PD0325901 (white bars). Results are obtained from 4 independent experiments. Results are represented as mean of MRFIs (or as % of CD16^+^ cells) ± SEM. **, *p* < 0.01*, *p* < 0.05; by Student's *t* test.

We investigated the effect of BRAF-i and MEK-i on the expression of activating NK receptors/co-receptors that have been shown to play a major role in NK-mediated melanoma cell lysis (i.e. NKp46, NKp30, NKG2D and DNAM-1) [34–36]. NK cells isolated from healthy donors were stimulated, in the presence of either BRAF-i (PLX4032) or MEK-i (PD0325901), with IL-2, IL-15 or the combination of IL-15/IL-18. We have chosen this cytokine combination since IL-18 signaling potentiates NK cell effector function by synergizing with common γ chain cytokines [37]. In addition, it has been shown that peripheral blood NK cells are very rapidly and significantly activated with the combination of IL-15 and IL-18 [38]. The oncogene-targeting drugs were used at a concentration of 10 μM (non toxic for NK cells, see Figure 1) because most melanoma cell lines, displaying BRAF^V600^ mutations, are susceptible to PLX4032 in a range between 1μM and 10μM (Figure S1). The surface expression of activating receptors was analyzed by flow cytometry both in freshly isolated and cultured NK cells (3 days culture with cytokines either in the absence or in the presence of the drugs). In agreement with previous data, NK cells cultured in IL-2 or IL-15 displayed an increased expression of NKp30 and NKG2D. The activation marker CD69 (used as control) was expressed de novo. In the presence of PD0325901 the expression of NKp30, NKG2D and CD69 was markedly lower than in control NK cells, while PLX4032 had virtually no effect. The surface density of NKp46 and DNAM-1 was virtually unchanged. Also the expression of Killer cell Ig-like receptors (KIRs) (including KIR2DL1/S1, KIR2DL2/L3/S2, KIR3DL1/S1) and CD94/NKG2A was not modified in the presence of the various inhibitors (data not shown). NK cells cultured with IL-15/IL-18 displayed a lower expression of NKp30 and NKG2D as compared to NK cells cultured with IL-2 or IL-15 alone. The presence of either BRAF-i or MEK-i did not modify the levels of expression of NKp30 and NKG2D in IL-15/IL-18 NK cells, possibly due to the low levels of expression of these activating receptors. Of note, CD69 was highly expressed by IL-15/IL-18 NK cells also in the presence of PD0325901 (Figure 2A).

Finally, the Fc-γ receptor CD16 was similarly expressed in NK cells cultured in IL-2 or IL-15 either alone or in the presence of the drugs. On the other hand, NK cells cultured with IL-15/IL-18, either alone or in the presence of PLX4032 displayed a reduced expression CD16. While PD0325901- treated IL-15/IL-18 NK cells maintained the expression of CD16 (Figure 2A).

The morphology of NK cells exposed to the drugs was analyzed by light microscopy (Figure 2A, photos on the right). NK cells treated with cytokines and BRAF-i exhibited morphological characteristics of activated NK cells (i.e. presence of small cell clumps). When NK cells were treated with IL-2 or IL-15 in the presence of MEKi, they displayed the morphologic characteristic of resting cells (absence of cell clumps), whereas with IL-15/IL-18 cells were grouped in clumps. We analyzed the expression of cellular adhesion molecules (CAM), CD54/ICAM-1, CD58/LFA-3, CD18 and CD2 in MEKi-treated NK cells. NK cells cultured with IL-15/IL-18 in the presence of PD0325901, expressed CD54/ICAM-1 at a higher density than drug-treated NK cells cultured with IL-2 or IL-15 alone. In addition, a trend toward an increase of CD58/LFA-3 and CD18 expression is observed in PD0325901-treated IL-15/IL-18 NK cells, while the percentage of CD2 expressing NK cells did not change (Figure S2).

Statistical analysis confirmed that MEK-i, but not BRAF-i, downregulated the expression of NKp30, NKG2D and CD69 in NK cells cultured with IL-2 or IL-15 alone, while such effect was not detected in NK cells cultured with IL-15/IL-18. In addition, PD0325901, but not PLX4032, prevented CD16 downregulation induced by IL-15/IL-18 (Figure 2B).

### Effect of MEK-i and BRAF-i on cytokine-induced NK cell proliferation

**Figure 3:**
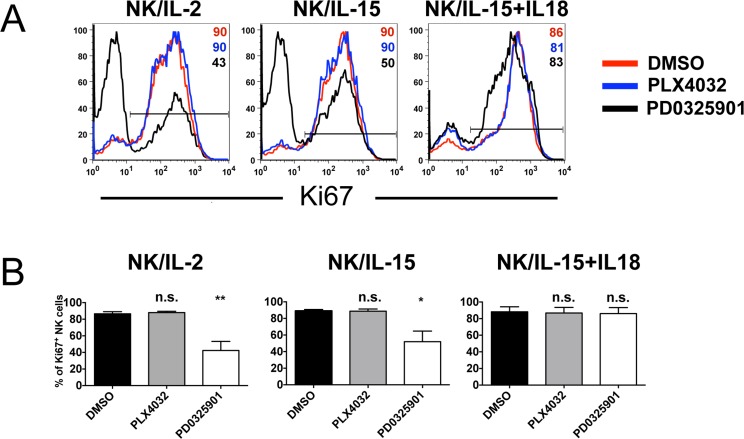
Effect of BRAF-i and MEK-i on NK cell proliferation Proliferative responses of NK cells isolated from 4 healthy donors cultured for 6 days with IL-2, IL-15 or IL-15/18 either in the absence (DMSO) or in the presence of PLX4032 or PD0325901 (10 μM). **A.** Representative flow cytometric analysis showing proliferating NK cells either untreated (red profiles) or exposed to PLX4032 (blue profiles) or PD0325901 (black profiles) expressing Ki67 are shown. Numbers indicate the percentages of proliferating cells. **B.** Statistical analysis of NK cell proliferation induced by cytokines in the absence or in the presence of BRAF-i (PLX4032) or MEK-i (PD0325901). Data are shown as means ± SD. Data are representative of 4 independent experiments. **, *p* < 0.01*, *p* ≤ 0.05; n.s., not significant by two tailed paired Student's *t* test.

We next analyzed the effect of BRAF-i and MEK-i on the cytokine-induced NK cell proliferation. In these experiments, NK cells were cultured with IL-2, IL-15 or IL-15/IL-18 either in the absence or in the presence of inhibitors. After 6 days, the expression of Ki-67, a cell-cycle-associated antigen exclusively expressed in proliferating cells, was assessed (Figure 3A). BRAF-i (PLX4032) had no effect on NK cell proliferation, whereas MEK-i (PD0325901) strongly inhibited proliferation of NK cells activated with IL-2 or IL-15. Of note, MEK-i did not reduce the proportion of proliferating NK cells in the presence of IL-15/IL-18. Statistical analysis confirmed the data (Figure 3B).

### Effect of MEK-i and BRAF-i on NK cell cytoxicity and cytokine production

**Figure 4:**
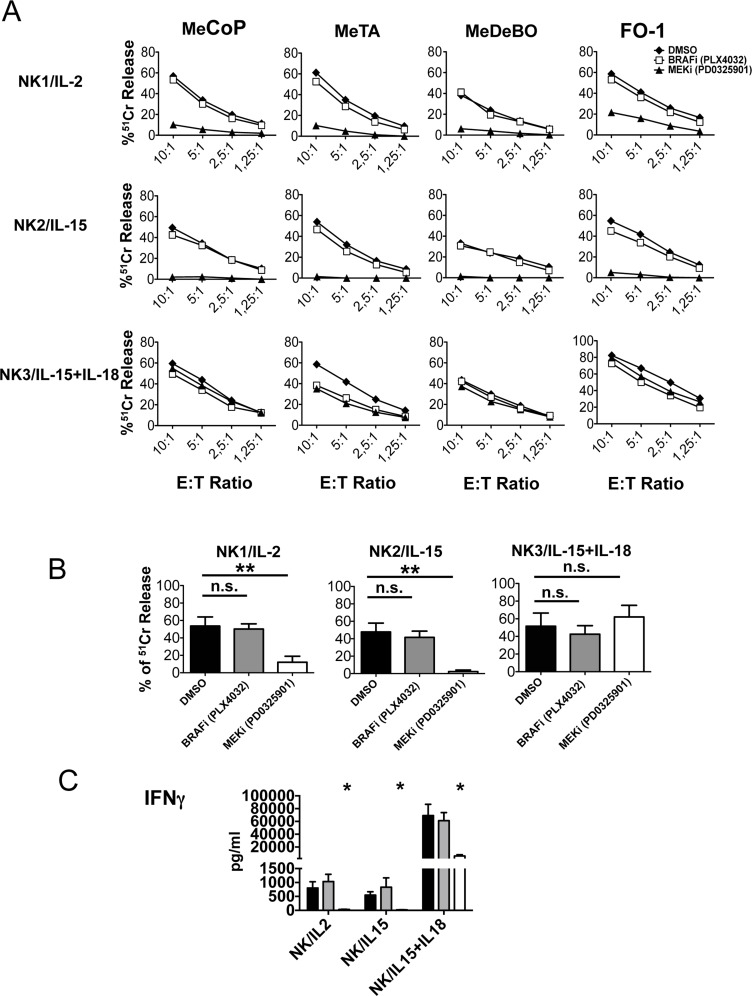
Effect of BRAF-i and MEK-i on NK cell function **A.** Cytotoxic activity of untreated or treated NK cells cultured for 3 days with the indicated cytokines against different melanoma cell lines (MeCoP, MeTA, MeDeBO, FO-1). Results of a representative experiment out of 3 performed are shown. Data represent the percentage of lysis mediated by NK cells. **B.** Overall cytolytic activity mediated by NK cells cultured with different cytokines in the absence (black bar) or in the presence of PLX4032 (gray bars) or PD0325901 (white bars) against the 4 melanoma cell lines indicated in panel A. The E:T ratio was 10:1. Bars represent means ± SD obtained from 3 independent experiments. **, *p* ≤ 0.01; n.s., not significant by two tailed paired Student's *t* test. **C.** IFNγ release by NK cells cultured with IL-2, IL-15 or IL-15/IL-18 either in the absence (black bar) or in the presence of PLX4032 (gray bars) or PD0325901 (white bars). Results are represented as means ± SEM obtained from 4 independent experiments. *, *p* < 0.05 by two tailed paired Student's *t* test.

NK cells, cultured with IL-2, IL-15 or IL-15/IL-18 and in the presence of either BRAF-i or MEK-i, were analyzed for their ability to kill different melanoma cell lines including MeCoP, MeTA, MeDeBO and FO-1. While BRAF-i had no substantial effect, MEK-i sharply reduced tumor cell lysis mediated by IL-2- and IL-15-activated NK cells. On the other hand, NK cells cultured with IL-15/IL-18 maintained their cytolytic activity against melanoma targets in the presence of both inhibitors (Figure 4A). Statistical analysis confirmed the significance of these data (Figure 4B). Statistical significance was maintained at different effector-to-target ratios (5:1, 2.5:1 and 1.25:1) (not shown).

We next assessed the effect of different concentrations of PD0325901 on NK cells cultured with IL-2, IL-15 or IL-15/IL-18. To this end, NK cells were exposed for 3 days to different MEK-i concentrations (10μM, 1μM and 0.1μM) and then analyzed for CD69 expression and anti-tumor killing capability (Figure S3). Even in the presence of low drug doses (i.e. 1μM and 0.1μM), NK cells cultured with IL-2 or IL-15 displayed a markedly lower expression of CD69 than control NK cells (Figure S3, panel A). Also their killing capability was decreased in a dose dependent manner as compared with untreated cells (Figure S3, panel B). On the other hand, when NK cells were cultured with IL-15/IL-18, PD0325901 only marginally affected the expression of CD69. In addition, the cytolytic activity of IL-15/IL-18 cultured NK cells was unmodified by PD0325901, regardless of the concentration used (Figure S3, panel B).

In order to investigate whether IFN-γ production was affected by MEK-i and BRAF-i, NK cells were cultured for 3 days with IL-2, IL-15 and IL-15/IL-18 in the presence or absence of the drugs. In all NK cell cultures containing BRAF-i, IFN-γ was produced in amounts comparable to controls. In contrast, IFN-γ production was abolished in IL-2- or IL-15-cultured NK cells in the presence of MEK-i. NK cells cultured with IL-15/IL-18 were able to release higher amounts of IFNγ (as compared to IL-2 or IL-15-cultured NK cells). Of note, PD0325901 impaired but not completely abolished the IL-15/IL-18-induced IFN-γ production (Figure 4C).

Finally, given that combined inhibition of BRAF/MEK is associated with improved clinical outcomes among patients with BRAF V600-mutated metastatic melanoma [10], we further assessed whether the addition of IL-18 to IL-15 was also able to rescue NK suppression induced by BRAF/MEK co-inhibition. Thus, NK cells cultured with IL-15/IL-18 and exposed simultaneously to both drugs were analyzed for their anti-tumor activity and proliferative capability (Figure S4). The NK-mediated cytotoxicity against melanoma cell lines (Figure S4, panels A and B), as well as cytokine-induced proliferation (Figure S4 panel C), was not impaired.

### Both MEK-i and BRAF-i are compatible with protocols of NK-based adoptive immunotherapy

**Figure 5:**
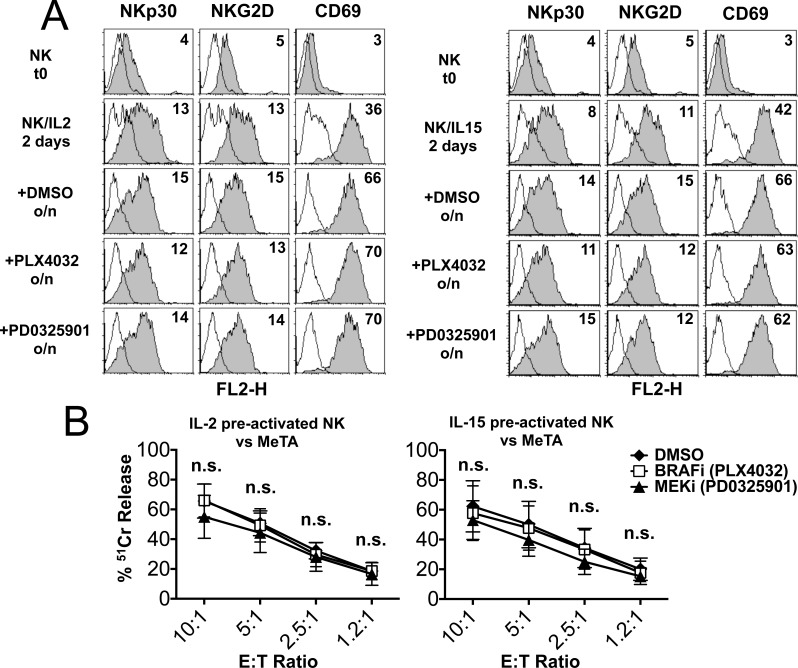
Effect of BRAF-i and MEK-i on IL-2- or IL-15- pre-activated NK cells Phenotypic and functional analysis of NK cells activated for 2 days with IL-2 or IL-15 (pre-activated NK cells) and then treated overnight (o/n) with PLX4032, PD0325901 or DMSO as control. **A.** Immunofluorescence analysis was done on freshly isolated NK cells (t0) and on cytokine pre-activated NK cells (day 2) exposed to the indicated drugs. Markers expression was analyzed with mAbs to the indicated molecules (filled gray histograms). White histograms represent negative controls. Numbers indicate the MRFI for each receptor. Results of a representative experiment out of 3 performed are shown. **B.** Cytolytic activity of pre-activated NK cells (2 days) either untreated (DMSO) or treated o/n with the drugs against a melanoma cell line (MeTA). Data represent the percentage of lysis by untreated or treated NK cells. Results are represented as means ± SEM obtained from 3 independent experiments. n.s., not significant by two tailed paired Student's *t* test.

In the experiments described above, PB NK cells were cultured with IL-2, IL-15 or IL-15/IL-18 and the inhibitors were added at the beginning of the culture. Since IL-2- and IL-15- pre-activated NK cells may be used in protocols of adoptive immunotherapy in cancer patients, we further investigated the effect of BRAF-i and MEK-i on NK cells that had been pre-activated for 2 days with IL-2 or IL-15. To this end, NK cells isolated from healthy donors were cultured in the presence of IL-2 or IL-15 for 2 days and further treated overnight (o/n) with either BRAF-i or MEK-i. The phenotypic analysis was focalized on the expression of NKp30, NKG2D and CD69, as it was markedly impaired in freshly isolated NK cells cultured with IL-2 or IL-15 in the presence of PD0325901 (see above). As shown in Figure 5A, neither BRAF-i nor MEK-i had any effect on the expression of NKp30 NKG2D, and CD69. In addition, the cytolytic activity of IL-2 or IL-15-pre-activated NK cells was not impaired in the presence of both drugs (Figure 5B and Figure S6). On the other hand, PD0325901 was able to inhibit the cytotoxicity of freshly isolated NK cells cultured overnight (o/n) in the presence of IL-2 or IL-15 (Figure S5). These data suggest that both PLX4032 and PD0325901 may be combined with NK cell-based adoptive immunotherapeutic strategies without affecting the efficacy of IL-2 or IL-15-pre-activated NK cells.

## DISCUSSION

In this study we show that while the BRAF-i PLX4032 had no significant effect, the MEK-i PD0325901 strongly inhibited the surface expression of the main activating receptors and the anti-tumor activity of freshly isolated NK cells cultured with IL-2 or IL-15. Importantly, no functional inhibition occurred in NK cells exposed to a combination of IL-15 and IL-18. While in IL-15/IL-18 untreated and in BRAFi-treated NK cells only a small fraction of cells expressed CD16, the large majority of IL-15/IL-18 MEKi-treated cells expressed this marker, thus allowing MEKi-treated NK cells to preserve their capability of mediating antibody-dependent cell cytotoxicity (ADCC). Interestingly, both inhibitors had no effect when added to NK cells pre-activated with IL-2 or IL-15, thus offering an important clue for the development of novel therapeutic strategies combining BRAF/MEK inhibitors with adoptive NK cell therapy.

The identification of activating somatic mutations in serine-threonine protein kinase BRAF (BRAF^V600E^) in 50% of patients with advanced melanoma offered the opportunity to develop oncogene-targeted therapies for this tumor. To date, different kinase inhibitors that target the constitutive up-regulated MAPK pathway have been generated and applied in clinical trials in the cure of melanoma. In particular, the administration of the BRAF^V600^-i Vemurafenib (PLX4032) or the MEK-i Trametinib resulted in an increased overall survival as compared to conventional chemotherapy [39–41]. The anti-tumor effect achieved with BRAF-i or MEK-i is primarily exerted on melanoma cells, resulting in marked shrinkage of tumor lesions consequent to apoptotic cell death. However, despite the encouraging response rates (ranging from 20 to 50%) and the impact on patient survival, in most cases responses to BRAF-i or MEK-i are transient [42]. Thus, the development of drug resistance eventually leads to tumor relapses.

Different immunotherapeutic approaches, including IL-2 therapy, and adoptive T cell transfer achieved promising results with a substantial clinical benefit at least in a fraction of melanoma patients [43]. Thus, it is conceivable that protocols that combine immune-based therapies with kinase inhibitors may result effective and thus need to be further explored. Along this line, recent evidences suggested that inhibition of the MAPK pathway in melanoma cells may result in the block of the production of tumor-derived immunosuppressive or proangiogenetic factors including IL-6, IL-10 and VEGF that are essential for cancer immune evasion and growth [44]. Other studies suggested that BRAF inhibition leads to increases of tumor infiltrating T cells in melanoma and induces the up-regulation of the melanoma differentiation antigens (MDA) in tumor cells [12]. These data support the notion that BRAF-targeted therapies may be used in association with T cell-based immunotherapy. Notably, however, a recent assessment of infiltrating T cells showed that they are characterized by an “exhausted phenotype” as revealed by an increased expression of PD-1 and its immunosuppressive ligand PD-L1 [45].

Also NK cells may represent powerful effectors against tumor as indicated by recent studies in patients with high-risk leukemia [46]. These studies provided clear evidence for a major role of NK cells in the cure of these malignancies in the haploidentical Hematopoietic Stem Cell transplantation setting. NK cells (derived from donor's CD34^+^ precursors) that express KIRs, mismatched with their HLA ligands in the donor *versus* recipient direction, clear leukemia blasts residual after the conditioning regimen, thus preventing leukemia relapses. In addition, a novel approach based on the depletion of TCR α/β^+^ T cells and B cells in which fresh alloreactive NK cells are infused together with CD34^+^ cells supports the notion that also fresh NK cells may represent suitable effectors against leukemia. Clinical results strongly support the notion that NK cells may indeed represent a powerful tool in tumor immunotherapy. Thus, it is conceivable that NK-based immunotherapy may be adopted also for the therapy of solid tumors, in particular melanoma.

Melanomas frequently lose the surface expression of MHC class I molecules thus acquiring resistance to MHC-restricted recognition by conventional T cells. This, together with the frequent expression on melanoma cells of ligands recognized by major activating NK receptors (including MICA/B, ULBPs, PVR, Nectin-2 and B7H6) [34–36, 47], suggests that NK cells may indeed represent important effectors against this tumor.

Thus, our study was designed to analyze whether different inhibitors of the MAPK pathway could interfere with the NK cell function. Indeed, we found that the MEK-i PD0325901 could sharply inhibit both cytolytic activity and NK cell proliferation in response to IL-2 or IL-15. On the other hand, the specific kinase inhibitor PLX4032 that targets mutated BRAF did not significantly alter IL-2- and IL-15-induced NK cell proliferation and cytolytic activity. Notably, IL-15/IL-18 cytokine combination spared the cytotoxic activity and proliferation of NK cells exposed to MEK-targeted agents. Thus, our data suggest that IL-15 and IL-18 cytokines could be utilized in combination with BRAF/MEK inhibitors to sustain and/or activate NK cell anti-tumor potential *in vivo*.

Although the intracellular mediators involved in the regulation of NKG2D ligands expression are still unknown, preliminary data would indicate that the MEK1/ERK, p38 MAPK, PI3K/Akt signaling pathways are involved in MICA and MICB up-regulation in cancer [48, 49]. These results suggest that BRAF-i and MEK-i may influence cancer susceptibility to NK cells. Opposite data have been reported regarding the susceptibility to NK cell-mediated killing of tumor cells resistant to kinase-target drugs [50, 51]. *In vitro* data would suggest that during the acquisition of BRAF-i resistance, tumor cells develop cross-resistance to NK-mediated lysis [51]. However, it has been shown that thyroid tumor cells, characterized by a constitutive activation of the MAPK pathway (due to the mutations of oncogenes such as RAS, BRAF, and RET/PTC), are more susceptible to NK cell-mediated killing. Notably, these BRAF-mutated tumor cells express the NKG2D ligands MICA/B at a higher level than unmutated tumors as a consequence of the activation of the MAPK pathway [50]. Moreover, a common mechanism by which tumor cells may by-pass the inhibitory effect exerted by RAF is the re-activation of the MAPK pathway by MEK and ERK signaling [52]. These data suggest that tumor cells resistant to BRAF-i may overexpress ligands recognized by activating NK receptors as a consequence of the MAPK pathway activation, thus becoming more sensitive to NK-mediated lysis.

In conclusion, our data also indicate that both the MEK-i PD0325901 and the BRAF-i PLX4032 do not exert any inhibitory effect on IL-2 or IL-15 pre-activated NK cells, thus suggesting that NK cell-based immunotherapy, used in combination with BRAF/MEK inhibitors, may represent an novel promising strategy in the treatment of melanomas.

## MATERIALS AND METHODS

### Monoclonal antibodies

The following monoclonal antibodies (mAbs) were used in this study: PE-conjugated anti-NKp46 (clone BAB281/IgG1, PN IM3711), PE-anti-NKp30 (clone Z25/IgG1, PN IM3709), PE-anti-NKp44 (clone Z231/IgG1, PN IM3710), PC5-anti-CD56 (clone N901-NKH-1/IgG1, PN A79388) were from Beckman Coulter. PE-anti-CD16 (clone VEP13/IgM, 130-091-245), PE-anti-NKG2D (clone BAT221/IgG1, 130-092-672), PE-anti-CD69 (clone FN50/IgG1, 130-092-160), FITC-anti-CD3 (clone BW264/56/IgG2a, 130-080-401) were from Miltenyi Biotec. PE-anti-DNAM-1 (clone 11A8/IgG1, 338306) was from Biolegend. Alexa Fluor 647-Anti-human Ki-67 (clone B56/IgG1, 558615) was from BD.

### Flow-cytofluorimetric analysis

For cytofluorimetric analysis cells were stained with the appropriate labeled mAbs. To compare the surface densities of NK receptors among NK cells cultured in the presence or in the absence of BRAF-i or MEK-i the mean ratio fluorescence intensity (MRFI) was calculated; that is the ratio between the mean fluorescence intensity (MFI) of cells stained with the selected mAb and the MFI of unstained cells. Data analyses were performed using FlowJo software (TreeStar Inc.).

### NK cell isolation and culture

NK cells were isolated from peripheral blood mononuclear cells (PBMCs) using the Human NK Cell Enrichment Cocktail-RosetteSep (StemCell Technologies Inc., 15065). Only populations displaying more than 95% of CD56^+^ CD3^−^ CD14^−^ NK cells were selected for the experiments. The isolated NK cells were cultured for 3 or 5 days in complete medium: RPMI 1640 (Lonza, 12-167F) 10% plus AB serum (Biowest, S4190-100), 1% penicillin/streptomycin (Lonza, 17-602E), 1% glutamine (Lonza, 17-605E) with 100U/ml IL-2 (Proleukin, Novartis), with 20ng/ml IL-15 (Miltenyi Biotech, 130-093-955), or with 20ng/ml IL-15 plus 0.1μg/ml IL-18 (MBL, B001-5) [53], in the presence or in the absence of BRAF-i (PLX4032, S1267) or MEK-i (PD0325901, S1036) (Selleckchem) dissolved in DMSO. All experiments were performed in accordance with approvals from the Liguria Regional Committee.

### Apoptosis analysis

NK cells were cultured for 5 days in complete medium with 100U/ml IL-2 and treated with increasing concentrations of BRAF-i (PLX4032) or MEK-i (PD0325901) dissolved in DMSO or with DMSO as negative control. The cells were transferred to FACS tubes and stained with Annexin V and propidium iodide (PI) following the manufacturer's instructions (Immunostep, ANXVKF-100T) and analyzed by flow cytometry.

### MTT assay

NK cells (2×10^6^/ml) and melanoma cells (5×10^4^-2.5×10^4^/ml) were cultured in 96-well U-bottom plates in complete medium either in the presence or in the absence of different concentrations of PLX4032. NK cell culture medium was supplemented with IL-2 (100U/ml). After 5 days, 10μl of MTT reagent 5mg/ml (Sigma Aldrich, 57360-69-7) was added to each well and the plates were incubated for 4 hours at 37°C in a CO_2_ incubator. After incubation, 100μl of medium was aspirated and 100μl of Lysis Buffer (10% SDS and 0.01M HCL in H_2_O) was added to each well. The absorbance of each sample was measured at 570nm using a microplate reader.

### NK cell proliferation

NK cells were plated in 96-well plates (1×10^6^/ml) and cultured in complete medium with IL-2, IL-15 or IL-15/IL-18 either in the presence or in the absence of the indicated drugs. After 6 days, NK cell proliferation was assessed using a Ki-67 staining protocol. Intranuclear staining was carried out using the FoxP3 permeabilization solution kit according to the manufacturer's instructions (eBioscience, BMS00-5223-56). Ki-67 mAb was added and cells were incubated at 4°C for 30 minutes. Finally, the cells were washed twice, re-suspended in 1ml of 1% fixation buffer, and analyzed by flow cytometry (FACSCalibur, BD).

### Cytolytic assays and cytokine production

Fresh or activated NK cells were cultured in complete medium in the presence or in the absence of BRAF-i (PLX4032) or MEK-i (PD0325901) were tested for cytolytic activity in a 4-hour ^51^Cr-release assay against four human melanoma cell lines (i.e MeCoP, MeTA, MeDeBO and FO-1). The E:T ratios are indicated in the figures. The supernatants derived from both untreated and drug-treated NK cells were harvested, centrifuged to remove any cells or debris, and frozen at −80°C until assayed. Production of IFNγ was measured from NK-derived supernatants with by commercial ELISA kits (ThermoFischer SCIENTIFIC, KHC4021C) following the manufacturers' instructions.

### Melanoma cell lines

Primary melanoma cell lines MeCoP, MeTA, MeDeBO, and MeOV were originated from metastatic lesions obtained in accordance with consent procedures (n.OMA09.001) approved by the Internal Ethics Board of the National Cancer Institute (IST, Genoa, Italy). Tissue specimens were processed for establishment of the cell lines as previously described [47]. FO-1 cell line was provided by Dr. Ferrone (New York Medical College). We confirmed and authenticated FO-1 cell line in our lab by PCR-SSP HLA class I typing (HLA-A25, -B08, -Bw6, and -Cw7) [54, 55].

### Statistical analysis

Statistical analyses were performed using the Prism software package (release 5.00; GraphPad Software). Statistical significance was evaluated by Student's t test. A P value of less than 0.05 (*), less than 0.01 (**), or less than 0.001 (***) was considered statistically significant.

## SUPPLEMENTARY MATERIALS FIGURES



## Abbreviations

ACT: adoptive cell therapy
ADCC: antibody-dependent cell-mediated cytotoxicity
BRAFi: BRAF inhibitors
IL: interleukin
KIRs: Killer cell Ig-like Receptors
MAPK: mitogen-activated protein kinase
MDA: melanocytes differentiation antigens
MEKi: MEK inhibitors
NK: Natural Killer
PB: Peripheral Blood
PI: propidium iodide
