# Supplementary material for: Cytokines can counteract the inhibitory effect of MEK-i on NK-cell function
Source: Oncotarget. 2016 Aug 22;7(38):60858–71. doi: 10.18632/oncotarget.11504 (PMC5308621; doi:10.18632/oncotarget.11504)
Supplement: Supplementary file 1 [file oncotarget-07-60858-s001.pdf]

Cytokines can counteract the inhibitory effect of MEK-i on NK-cell function

Supplementary Material

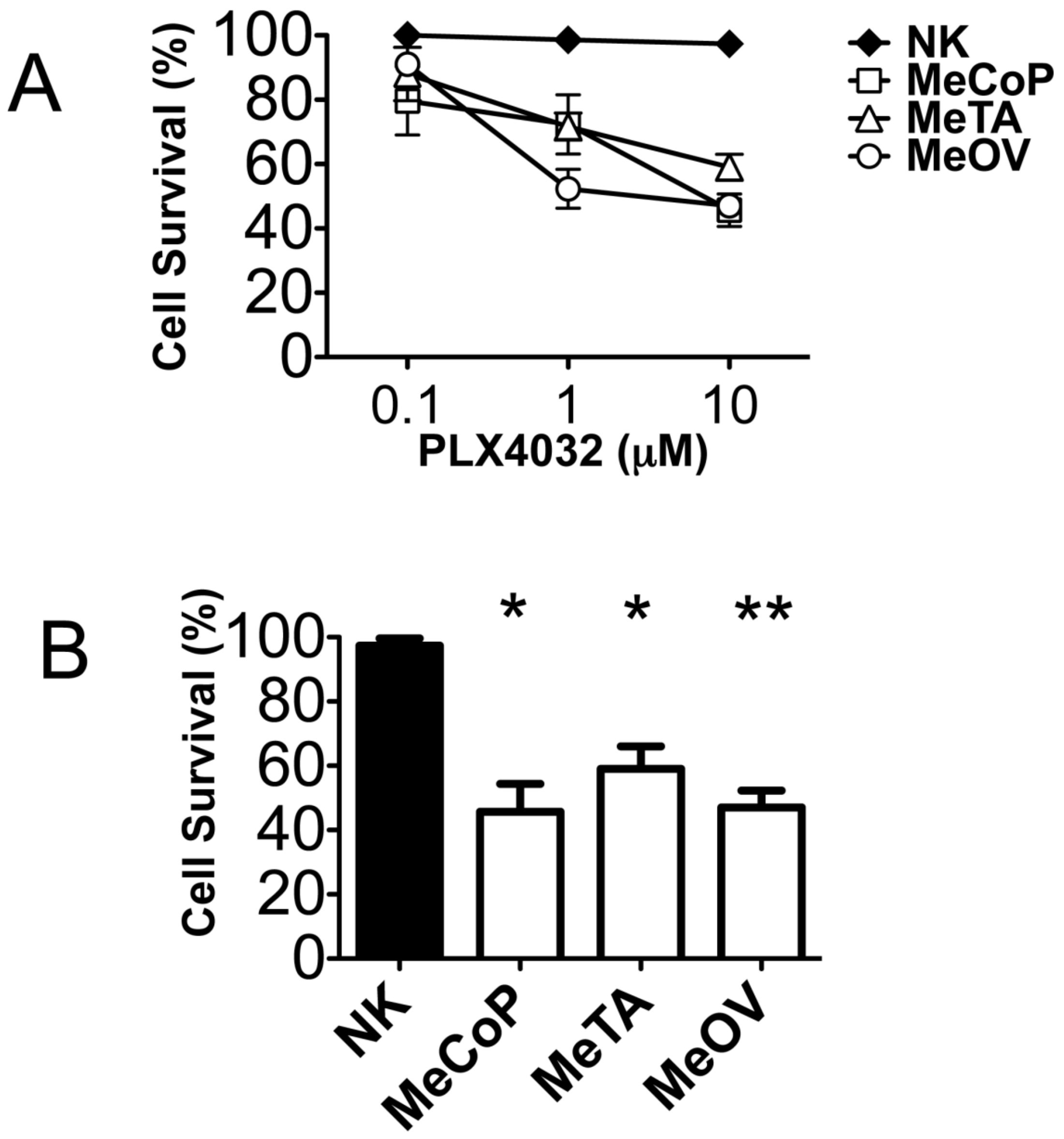

Figure S1. Survival of NK cells to different concentrations of PLX4032 that exert anti-tumor effect. NK cell and melanoma cell survival (evaluated by MTT test) upon treatment with different concentrations (10μM, 1μM and 0.1μM) of PLX4032. NK cells derived from healthy donors and 3 BRAF-mutated melanoma cell lines (MeCoP, MeTA, and MeOV) were cultured for 5 days in the presence of the drug. NK cells were cultured in medium with IL-2. ***(A)*** Percentage of NK or melanoma cell survival after treatment at the indicated drug concentrations. Results are obtained from 3 independent experiments. Each point represents mean ± SD. ***(B)*** Statistical analysis of NK

cell (black bar) or melanoma cell (white bars) survival in the presence of PLX4032 (10μM). Results are obtained from 3 independent experiments. Results are represented as mean of percentage of cell survival +/- SD. **, $p < 0.01$; *, $p < 0.05$, by two tailed paired Student's *t* test.

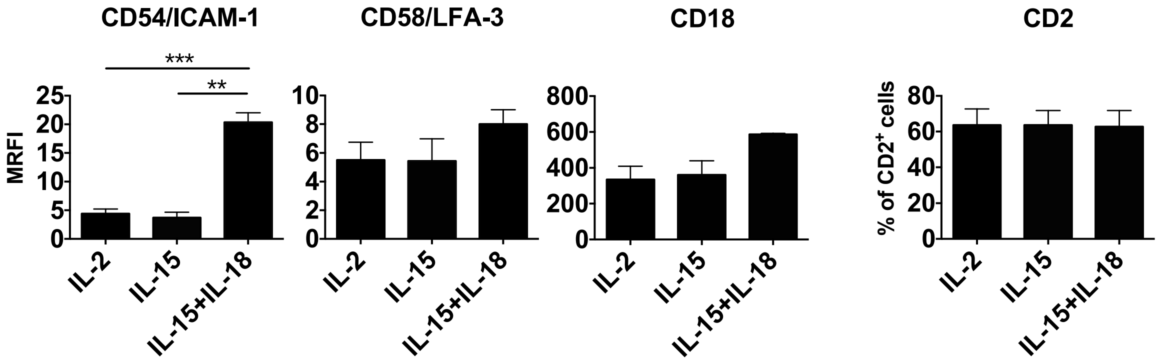

Figure S2. Expression of CD54/ICAM-1, CD58/LFA-3, CD18 and CD2 in MEKi-treated NK cells. The expression of the indicated markers was analyzed on NK cells cultured for 3 days with IL-2, IL-15 or IL-15/IL-18 in the presence of PD0325901 (10μM). Results are obtained from 3 independent experiments. Results are represented as mean of MRFIs (or as % of $CD2^{+}$ cells) ± SEM. ***, p< 0.001; **, p < 0.01, by Student's *t* test.

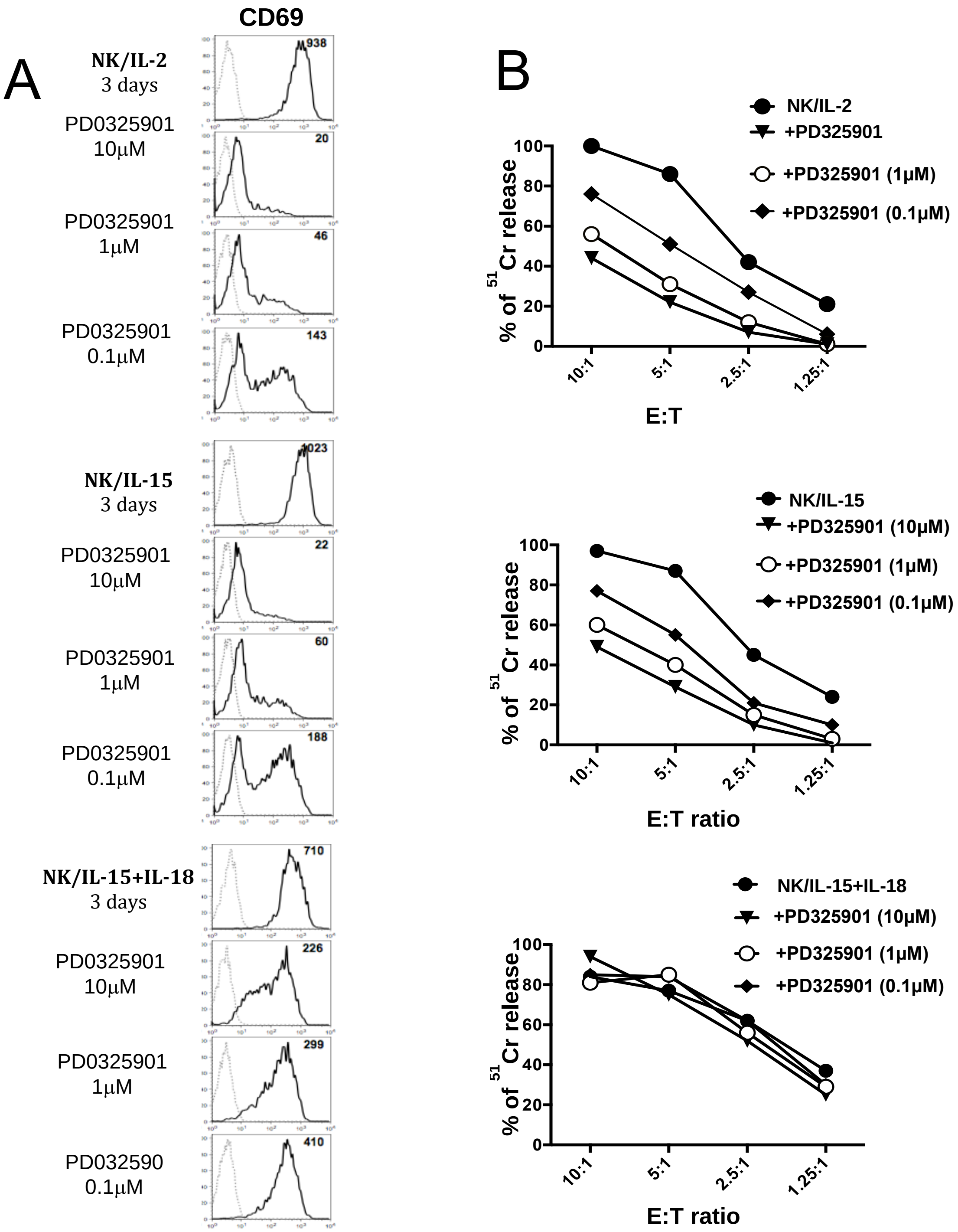

Figure S3. Effect of scalar concentrations of MEKi (PD0325901) on NK cells exposed to different cytokines. ***(A)*** CD69 surface expression in NK cells isolated from one representative healthy donor cultured for 3 days with IL-2, IL-15 or IL-15/IL-18 either in the absence or in the presence of various concentrations of PD0325901. NK cells cultured with cytokines in the presence of DMSO represent the negative controls. The expression of CD69 activation marker has been analyzed by flow cytometry (FACSCalibur, BD). Numbers indicate the MFI. Results of a representative experiment are shown. ***(B)*** Cytotoxic activity of untreated (DMSO) or PD0325901 treated NK cells cultured with the indicated cytokines against FO-1 melanoma cell line. Results of a representative experiment performed are shown.

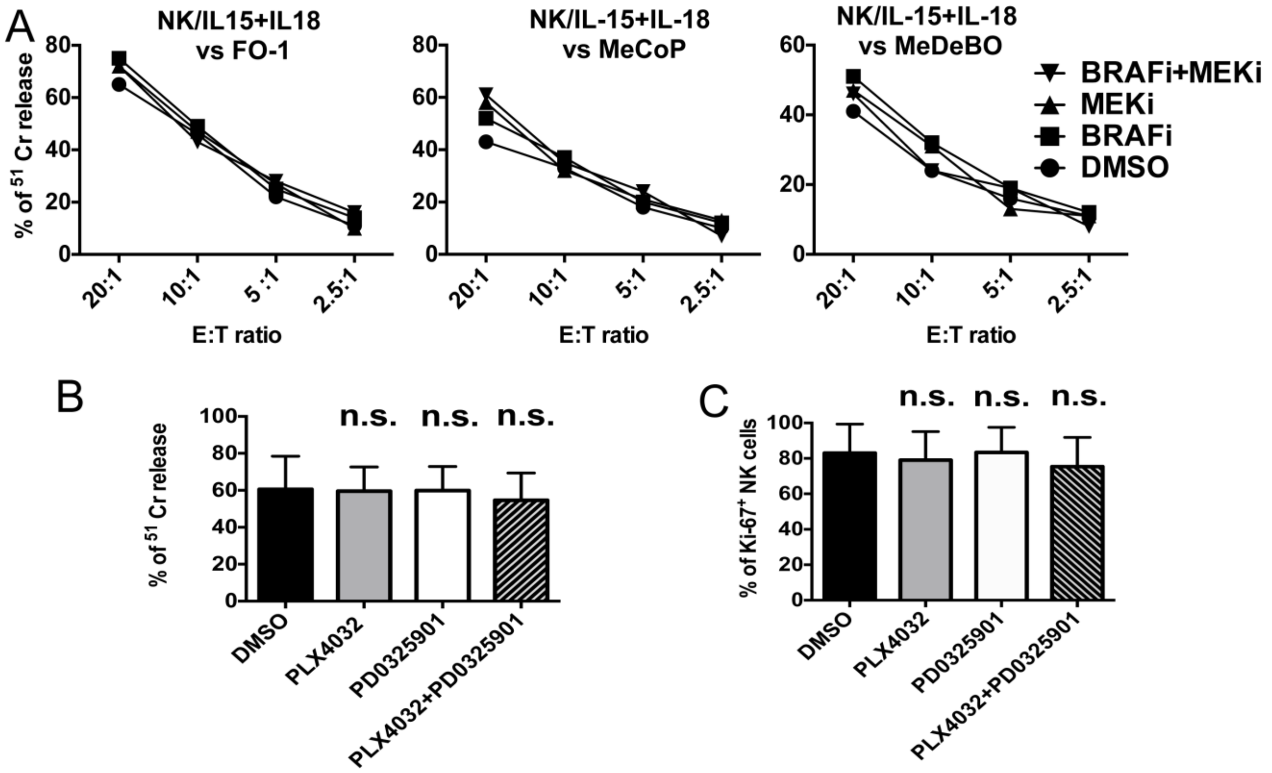

Figure S4. IL-15/IL-18 cytokine combination overcomes the inhibitory effect of MEK-i on NK cell function. ***(A)*** Cytotoxic activity of untreated (DMSO) or drug treated NK cells obtained from a healthy donor cultured with IL-15/IL-18 against 3 different melanoma cell lines. Results of a representative experiment performed are shown. ***(B)*** Statistical analysis of the percentage of lysis mediated by NK cells cultured with IL-15/IL-18 in the absence or in the presence of PLX4032, PD0325901 or PLX4032 plus PD0325901 (10μM) against melanoma cells. The E:T ratio was 20:1. Bars represent means ± SD obtained from 8 independent experiments. n.s., not significant by two tailed paired Student's *t* test. ***(C)*** % of Ki-67 expressing NK cells isolated from 3 healthy donors cultured for 6 days with IL-15/IL-18 either in the absence or in the presence of PLX4032, PD0325901 or PLX4032 plus PD0325901 (10μM). Data are shown as means ± SD. Data are representative of 3 independent experiments. n.s., not significant by two tailed paired Student's *t* test.

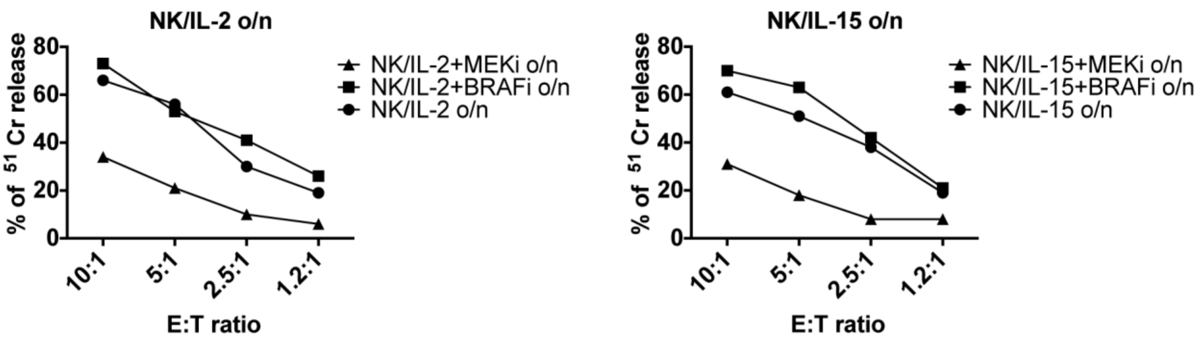

Figure S5. **Effect of BRAFi and MEKi on NK cells cultured o/n with IL-2 or IL-15**. Cytotoxic activity of freshly isolated NK cells cultured o/n with IL-2 or IL-15 in the absence or in the presence of PLX4032 (BRAFi) or PD0325901 (MEKi) (10µM) against FO-1 melanoma cell line. Results of a representative experiment out of 3 performed are shown.

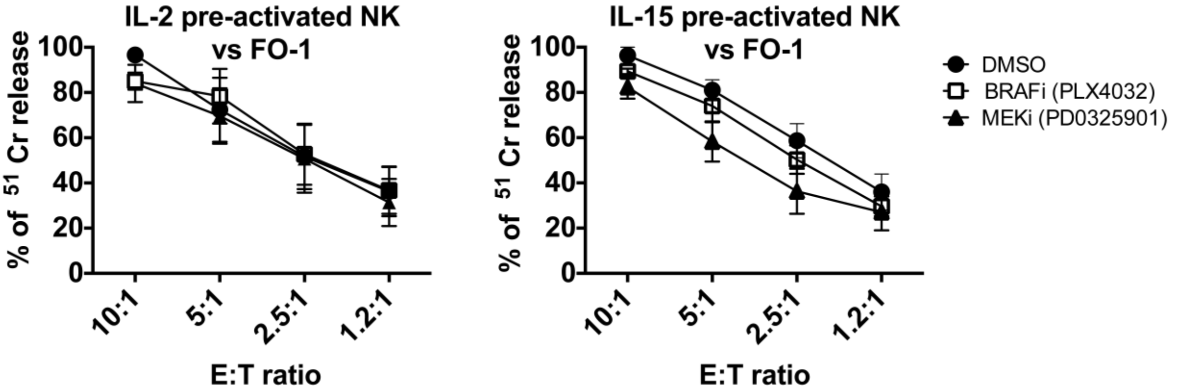

Figure S6. **Effect of BRAFi and MEKi on IL-2 or IL-15 pre-activated NK cells**. Cytolytic activity of pre-activated NK cells (2 days) either untreated (DMSO) or treated o/n with the drugs against FO-1 melanoma cell line. Data represent the percentage of lysis by untreated or treated NK cells. Results are represented as means ± SEM obtained from 3 independent experiments

Supplementary methods

Expression of adhesion molecules by NK cells and flow cytofluorimetric analysis.

The following mAbs were used: 14D12D2 (IgG1, anti-CD54/ICAM-1), TS2/9 (IgG1, anti-CD58/LFA-3), EA4 (IgG2a, anti-CD18/βLFA-1) and MAR206 (IgG1, anti-CD2). PE-conjugated anti-isotype goat anti-mouse mAbs were purchased from Southern Biotechnology Associated (Birmingham, AL, USA). To compare the surface densities of adhesion molecules among NK cells cultured with different cytokines in the presence MEK-i the mean ratio fluorescence intensity (MRFI) was calculated; that is the ratio between the mean fluorescence intensity (MFI) of cells stained with the appropriate mAb followed by PE-conjugated isotype-specific goat anti-mouse second reagent and the MFI of cells stained with PE-conjugated isotype-specific goat anti-mouse second reagent. Data analyses were performed using FlowJo software (TreeStar Inc.).
